# Neocytolysis: none, one or many? A reappraisal and future perspectives

**DOI:** 10.3389/fphys.2014.00054

**Published:** 2014-02-14

**Authors:** Angela Risso, Annarita Ciana, Cesare Achilli, Guglielmo Antonutto, Giampaolo Minetti

**Affiliations:** ^1^Department of Agricultural and Environmental Sciences, University of UdineUdine, Italy; ^2^Laboratories of Biochemistry, Department of Biology and Biotechnology, University of PaviaPavia, Italy; ^3^Department of Medical and Biological Sciences, University of UdineUdine, Italy

**Keywords:** neocytolysis, erythropoietin, red cell lifespan, red cell mass regulation, red cell senescence, microgravity, space flight, mountaineering

## Abstract

Neocytolysis is the hypothesis formulated to explain experimental evidence of selective lysis of young red blood cells (RBCs) (neocytes) associated with decreased plasma levels of erythropoietin (EPO). In humans, it appears to take place whenever a fast RBC mass reduction is required, i.e., in astronauts during the first days of spaceflight under weightlessness, where a fast reduction in plasma volume and increase in haematocrit occur. EPO plasma levels then decline and a decrease in RBC mass takes place, apparently because of the selective lysis of the youngest, recently generated RBCs (neocytes). The same process seems to occur in people descending to sea level after acclimatization at high altitude. After descent, the polycythaemia developed at high altitude must be abrogated, and a rapid reduction in the number of circulating RBCs is obtained by a decrease in EPO synthesis and the lysis of what seem to be young RBCs. *In vivo*, neocytolysis seems to be abolished by EPO administration. More recent research has ascribed to neocytolysis the RBC destruction that occurs under such disparate pathophysiologic conditions as nephropathy, severe obstructive pulmonary disease, blood doping, and even malaria anaemia. According to the theory, EPO's central role would be not only to stimulate the production of new RBCs in conditions of anaemia, as maintained by the orthodox view, but also that of a cytoprotective factor for circulating young RBCs. Why neocytes are specifically destroyed and how is this related to decreased EPO levels has not yet been elucidated. Changes in membrane molecules of young RBCs isolated from astronauts or mountain climbers upon return to normal conditions seem to indicate a higher susceptibility of neocytes to ingestion by macrophages. By limiting the context to space missions and high altitude expeditions, this review will address unresolved and critical issues that in our opinion have not been sufficiently highlighted in previous works.

## Introduction

The red blood cells (RBCs) of mammals are non-nucleated cells that spend in the circulation a limited amount of time, after which they are removed by the reticulo-endothelial system according to a species-specific type of kinetic. This results from the superimposition of random destruction (independent of cell age) and of a senescence process. The magnitude of the random component varies in different species and in different animals within the same species. It is very pronounced in mice and rats, less pronounced in pigs, rabbits and other mammalians, and almost absent in normal human RBCs, which are recognized as senescent and removed after 120 days of circulatory life (Clark, 1988; Landaw, 1988; Brovelli and Minetti, 2003). In various haematological disorders the destruction of poorly deformable or otherwise compromised RBCs occurs at a faster rate, with spleen as the main organ involved in the process, since splenectomy often alleviates the abnormal shortening of life-span of these cells (Landaw, 1988). On the other hand, it is believed that the spleen has only a modest role in the removal of normal senescent RBCs, which were shown to be phagocytosed almost exclusively in the bone marrow (Miescher, 1956; Marton, 1970; Clark, 1988; Landaw, 1988). The mass of RBCs (RBCM) circulating at each given time is the result of a dynamic balance between the destruction of old cells and the production of new ones, which derive from precursors of the erythroid lineage. The production of precursors is regulated, in its early phase, by various factors, among which are erythropoietin (EPO), that maintains cell vitality and transmits anti-apoptotic signals, and SCF (stem cell factor), a proliferation factor (Jacobs-Helber et al., 1997; De Maria et al., 1999). At later stages, primary erythroblasts proceed through their proliferation and differentiation programs, thanks to other factors, among which osteopontin appears to play a central role as a factor of proliferation and remodeling of the cytoskeleton (Kang et al., 2008). On the other hand, it is an accepted view that, under physiologic conditions, no mechanisms exist (or are not known), which are able to decrease the RBC life-span below an established, fixed value, which, in humans, amounts to the said 120 days (corresponding to the removal of approximately 1% RBCs per day).

## The neocyte and neocytolysis

The neologism “neocyte” was firstly adopted in the context of transfusion medicine in the late 70s, when possible improvements in the transfusion regimens for patients with haemoglobinopathies (particularly thalassemia) were under intensive study, and one approach was based on the infusion of “neocytes” (then defined as the 50% less dense circulating RBCs, which were also assumed to comprise the youngest RBCs) with higher survival rate, with the aim at reducing the frequency of transfusions, and thus iron overload, in these patients (Propper et al., 1980). More recent evidence has shown, however, that the infusion of neocytes is less advantageous than expected (Pisciotto et al., 1986), and it is nowadays not practiced on a large scale for the long-term treatment of patients (Forget and Olivieri, 2003).

**Figure 1:**
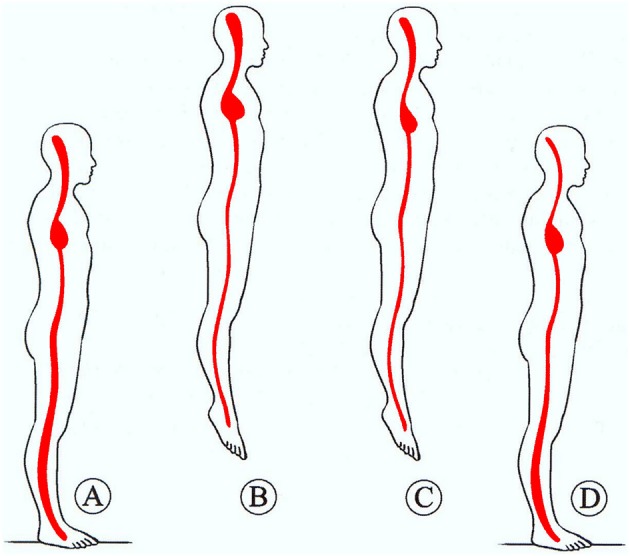
**Representation of central blood pooling in space**. Upon transition from normogravity **(A)** to microgravity **(B)** the blood of the peripheral vascular space shifts to central space, causing central acute plethora, accompanied by peripheral vessel constriction. An adjustment is obtained by reduction of PV and RBC mass through erythrolysis and EPO reduction **(C)** over the first days of spaceflight. Then, upon return to normogravity **(D)**, the normal redistribution of blood volume and the augmentation of blood fluid reduce the haematocrit (space anemia). From Charles et al. (1994).

The term “neocytes” has been adopted again, more recently and in an entirely different context, with the theory proposed by Alfrey and co-workers to explain the anaemic condition that affects astronauts after space flights (“space anaemia”) (Alfrey et al., 1997). In 1965, a study showed that in astronauts participating in orbital flights Gemini IV, V, and VII, a decrease in erythrocyte survival and in RBCM was occurring, due to erythrolysis of unknown cause (Fischer et al., 1967). On escaping the Earth's gravitational force the human organism experiences a reduction in total blood volume, plasma volume (PV) and, most importantly, of RBCM. The peripheral blood normally held in place by gravity, moves to central organs where a condition of acute plethora ensues. At the same time, a 20% reduction in PV takes place by redistribution in various compartments, thus inducing an increase in haematocrit (“pseudopolycythaemia”) (Watenpaugh, 2001; De Santo et al., 2005). In the following days, a drop in EPO levels is observed, along with a decrease in RBCM of 10–15%, that has been likened to a phlebotomy of 700 ml of blood (Figure 1). According to the neocytolysis hypothesis, the latter decrease occurs too rapidly to be only the result of combined suppression of erythropoiesis and continued, normal destruction of physiologically aged RBCs (occurring at a rate of less than 1% per day), but could be explained by the selective lysis of relatively young RBCs, the “neocytes.”

Neocytolysis would effect a finely-tuned and rapid regulation of the RBCM for a more efficient adaptation to mutated environmental conditions such as the following situations: in high-altitude dwellers (alpinists in mountaineering expeditions) returning to a normoxic environment at sea level; in anaemic uraemic patients requiring therapy with exogenous EPO (Rice et al., 1999); in patients with combined kidney and heart failure where a resistance to EPO treatment has been described to occur frequently (De Santo et al., 2005; van der Putten et al., 2008); and in a human model based on cycles of EPO injection and withdrawal (Rice and Alfrey, 2005). Moreover, the neocytolytic process could be relevant in other contexts as polycythaemia vera, blood-doping in sports, haemolytic anaemias (Mentzer et al., 1971), in patients with severe chronic obstructive pulmonary disease when subjected to oxygen therapy, where neocytolysis would occur upon therapeutic relieving of the hypoxic conditions (Harris and Epstein, 2001), and for the development of malaria anaemia, as recently proposed (Fernandez-Arias et al., 2013).

Comprehensive and stimulating reviews and commentaries have appeared that deal with neocytolysis, some from the same researchers who proposed the theory (Alfrey et al., 1997; Rice and Alfrey, 2000, 2005; Alfrey and Fishbane, 2007), some by others (Means, 1999; Harris and Epstein, 2001; De Santo et al., 2005; Handelman and Levin, 2010). We will limit this review to address some unresolved issues that in our opinion have not been sufficiently highlighted in previous works, and limit the focus on the experimental evidence obtained in space missions and high-altitude expeditions, the context where the theory was originally formulated, in particular in relation to the establishment of the link between EPO withdrawal and the removal of selected subpopulation(s) of RBCs.

## Experimental evidence of the neocytolytic process in space missions

A decrease in RBCM of variable amounts was a consistent finding in astronauts during space flights. It was originally imputed to oxidation-induced haemolysis or oxygen inhibition of erythropoiesis, because an atmosphere of pure oxygen was breathed aboard of Gemini or Apollo missions. It was however observed also in subsequent missions where the atmosphere was similar to air at sea level, pointing therefore to a gravity-dependent mechanism capable of altering RBC survival. Evidence of erytholysis under microgravity came from studies on Spacelab Life Sciences missions 1 and 2 (SLS-1 and SLS-2) (Udden et al., 1995; Alfrey et al., 1996a). Results showed unequivocally a reduction in RBCM, as measured, at landing, by radionuclide dilution techniques with ^51^Cr-labeled autologous RBCs and ^125^I-iodinated albumin. When data from two other missions (Spacelab-1 and Space Transport System 41-B) were also considered, RBCM reduction was linearly correlated with mission length, and the extrapolation to day zero of flight suggested a faster RBCM decrease during the first days of flight (Alfrey et al., 1996a). On the first day of flight, PV also decreased by a significant 17%, with respect to values measured on 2 pre-flight days, thanks to the egress of albumin-containing fluid from the vascular space (the reasons for this are by no means clear, see Watenpaugh, 2001), and the establishment of a condition of “acute plethora” ensued (Figure 1).

Peripheral blood parameters revealed increased RBC concentration in-flight, of approximately 20%, which was related to the concomitant decrease in PV (Alfrey et al., 1996a). The microhaematocrit measured in-flight by centrifugation did not increase, despite the fall in PV, and this was explained with the observed concomitant decrease in MCV (calculated from RBC concentration and haematocrit). The explanation was that the number of young cells, which are larger, was decreasing. Crew members were sampled as early as day two in-flight, and every other day until flight day 14, but it is not clear how early the MCV decrease began (Alfrey et al., 1996a). The question is not trivial because it could help distinguish between a true selective removal of young RBCs and a generalized decrease in cell volume or the shrinkage of a subpopulation of cells, perhaps still, but not necessarily, the youngest circulating cells. To the best of our knowledge, no other study published or commented on MCV values measured during space missions, except for a decrease from pre-flight values of 90.9 ± 3.1 to 89.3 ± 2.8 fL (statistically significant despite the small difference) recorded at landing in 11 astronauts of the International Space Station Expeditions 1–8 (2000–2004) (Smith et al., 2005), but these were long-term missions (128–195 days), where compensatory processes appear to reset the homeostatic mechanisms and alleviate even the reduction in RBCM (Tavassoli, 1982).

The other important measured haematological parameter was EPO, whose levels decreased significantly, by about 25%, but only at days 2–4 in-flight, to return normal at flight days 8–12 (Alfrey et al., 1996a). In the previous SLS-1 study of three crew members, EPO levels were constantly 31% lower in-flight, but the mission only lasted 9 days (Udden et al., 1995).

The ferrokinetic data obtained by injecting ^59^Fe 22 h or 72 h after launch for SLS-1 and SLS-2, respectively, and measuring the radiolabel in plasma and in RBCs, indicated that new RBC production in the bone marrow was not decreased from pre-flight values, and therefore this could not account for the magnitude of the decrease in RBCM (Alfrey et al., 1996b). New RBC production was not unexpected in SLS-1, where ^59^Fe was injected after 22 h in-flight, because, despite the transient EPO decrease, committed erythroid precursors that were conceived before the labeling will continue to mature and will be released in the circulation during flight. For this reason, in SLS-2 radioiron was injected after 72 h in-flight. In this case, too, results indicated a normal RBC production. But SLS-2 is the mission where EPO levels returned to normal at flight day 8. Nonetheless, Authors concluded that the decrease in RBCM must have originated from an accelerated RBC destruction with respect to the physiological value (approximately 1%/day).

Reticulocyte counts (expressed as percent of RBCs) were only mentioned in the text for SLS-1, and were found to be decreased, for each crew member, with an average for all subjects of 0.6% on landing, after 9 flight days, with respect to 1% before the flight. Unfortunately, reticulocyte counts were not given for SLS-2, which would have allowed refining the definition of neocytes as including or not the reticulocytes (see below). Available data on reticulocytes in two Gemini VII astronauts showed no change between pre-flight values and landing and 2 days post-flight values. However, changes in haematological parameters and RBCM in Gemini missions were in part (and demonstrably) determined by hyperoxia conditions (Fischer et al., 1967; Tavassoli, 1982). Reticulocyte data are available also for the four crew members of the 10-day Spacelab 1 mission in 1984 (Leach and Johnson, 1984). Strangely, the reticulocyte counts were significantly lower, by approximately 50%, already the day before launch, with respect to pre-flight values (taken 1 week before flight), and, after an apparent return to values only 17% lower than baseline (non-significantly) at flight day 1, they were significantly 50% lower than baseline again at flight day 7. Interestingly, only the latter statistical significance was taken into account, whereas the former was disregarded as the result of “such factors as stress and previous blood withdrawals” (incidentally, previous blood withdrawals should increase, not increase reticulocyte counts) (Leach and Johnson, 1984). Additional results of this work showed that EPO levels did not change, and that RBCM decreased by about 1%/day, concluding that inhibition of erythropoiesis was not the only cause of RBCM reduction. Subsequently, however, EPO concentrations were correctly measured, with a newly available radioimmunoassay, in samples from the same mission and were found to be significantly decreased from flight day 1 until landing of that relatively short space mission (7 days) (Leach et al., 1988).

Other confounding factors in erythrokinetic studies include the ferritin status during and after space flight. Serum ferritin has been found increased after many long-term and short-term space missions (Smith et al., 2001), an indication of increased iron storage resulting from the destruction of RBCs (neocytes?) taking place during flight. However, it is well known that increases in serum ferritin may also derive from inflammatory or general stress response. To overcome this limitation, iron content in ferritin should be quantified. Measurements of this kind in astronauts returning from long-term missions have revealed a decrease in ferritin saturation despite the increase in serum ferritin (Smith et al., 2001, 2005). On the other hand, the concentration of circulating transferrin receptors (which decreases with iron load) was decreased in one study (Smith et al., 2001) but not significantly decreased in a more comprehensive subsequent report (Smith et al., 2005).

## Random label method with ^51^Cr for evaluating RBC destruction

Stronger evidence for an increased destruction of young RBCs in space came from the ^51^Cr random labeling studies performed on six astronauts participating in the NASA shuttle missions SLS-1 and SLS-2. Here, autologous RBCs from six crew members from each mission were labeled with ^51^Cr and reinjected intravenously 21 days (SLS-1) or 12 days (SLS-2) before launch. Samples were then taken at intervals during the flight to determine the ^51^Cr specific activity, i.e., the counts per minute per millilitre RBCs. The percent change in ^51^Cr specific activity with respect to the value at time of injection was used as an estimate of RBC production and survival (Alfrey et al., 1996a).

**Figure 2:**
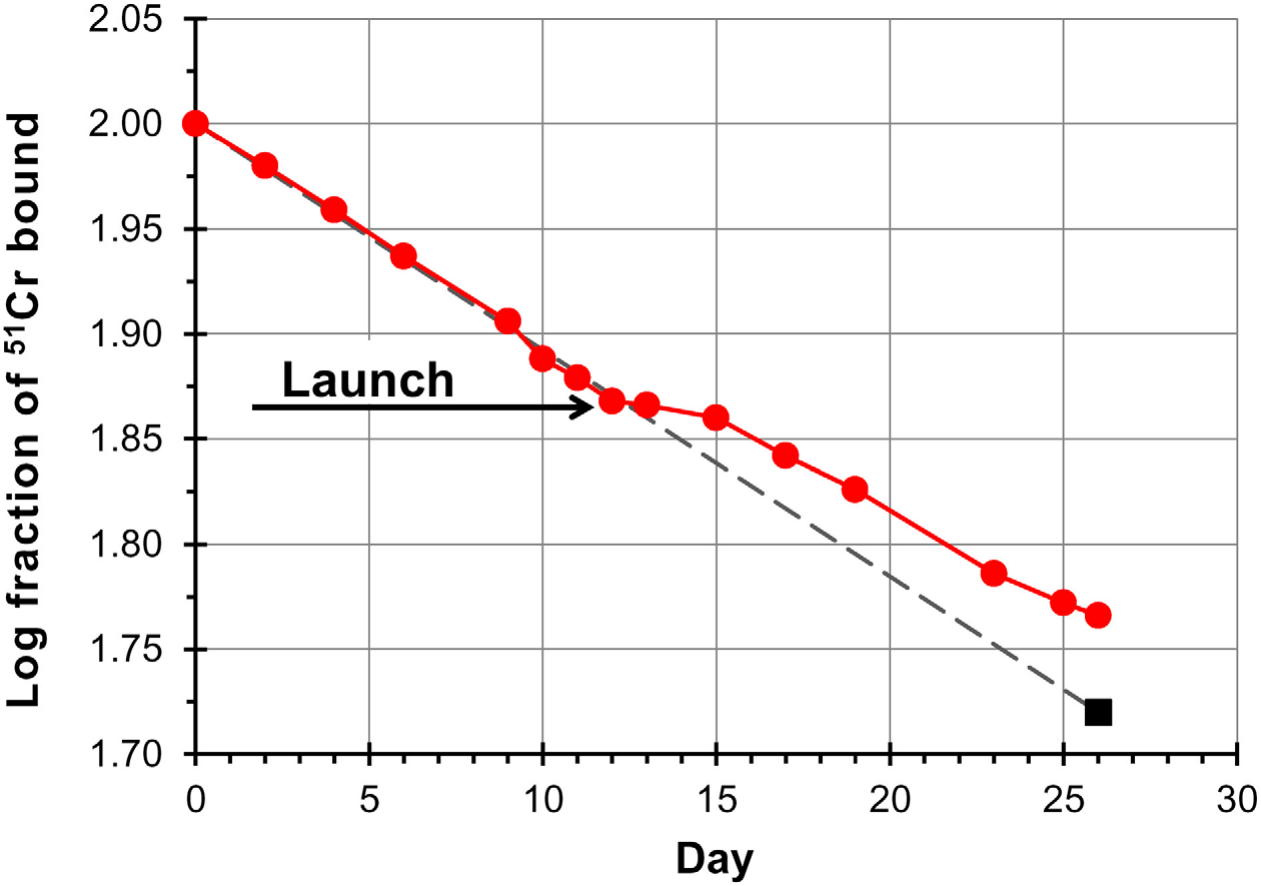
**Red cell survival on SLS-2 space mission**. Data points are a composite of results from three astronauts. Red cell survival is normal pre-launch whereas the inflection in the curve beginning at launch has been interpreted as the result of destruction of unlabeled erythrocytes (neocytes), and a consequent increase in the concentration of labeled cells. The last point (square symbol) is based on the measured chromium remaining, corrected for the cell mass measured on landing day. The fact that the trend line (dashed line) generated from preflight values transects this point demonstrates that older labeled red cells are removed from the circulation at the same normal rate in space as on Earth. Redrawn from Rice and Alfrey (2005).

Results showed that, after launch, the specific activity increased over that predicted had the astronauts remained on Earth. On landing day the mean difference was 6% more than predicted for the crew members, resulting from a much slower rate of change in specific activity in the first 4 days of flight with respect to that measured before flight (Figure 2). From data obtained in the SLS-2 mission the Authors concluded that the relative increase in ^51^Cr specific activity was the result of a selective decrease in unlabeled cells, those produced in the days before flight, after the radiolabeling, and during flight, and not only the consequence of decreased erythropoiesis (due to the decrease in EPO) combined with normal RBC destruction rate. Interestingly, when data obtained in the SLS-1 mission were originally published (Udden et al., 1995), it was concluded that erythropoiesis was partially inhibited because the ferrokinetic study with ^59^Fe revealed a lower incorporation of ^59^Fe into RBCs and that the observed 11% decrease in RBCM was the result of suppressed release of RBCs into the circulation (it was speculated that EPO could have a role in permitting the egress of RBCs for the bone marrow) together with the normal age-related destruction of circulating RBCs at the physiological rate of 1%/day (Udden et al., 1995). In fact, in the SLS-2 mission the decrease in RBCM was more pronounced, and could not be explained only by the physiological rate of RBC removal and the relatively modest suppression of erythropoiesis (Alfrey et al., 1996a).

The rate of disappearance of the ^51^Cr label from blood is determined by the rate at which new cells enter the circulation combined with the rate of elution of ^51^Cr from the labeled RBCs. The method and the data obtained in space missions are valid only under the assumption that the rate of elution of ^51^Cr from labeled RBCs is the same in space, under conditions of plethora and dramatic shifts in body fluids, as is on Earth. The Authors were conscious of this when they stated “If the rate of ^51^Cr elution is assumed to be unaffected by spaceflight… ” (Udden et al., 1995). However, as the rate of elution is known to vary amply, with an average of 1% per day under physiological conditions (Bentley et al., 1974), but with a much broader range of between 0.62 and 2.27% per day under pathological conditions (Cline and Berlin, 1963), one cannot but wonder what would be the situation under conditions of microgravity. In other words, a decrease in the steepness of the curves of ^51^Cr disappearance from the circulation (Alfrey et al., 1996a) could be also explained by a decreased elution of the ^51^Cr label taking place, for whatever reasons, as soon as the organism is exposed to microgravity conditions.

Another aspect that has not been sufficiently considered in the neocytolysis theory (but was contemplated in early studies, see Fischer et al., 1967) is the possible selective sequestration of young RBCs to other compartments, e.g., the spleen.

**Figure 3:**
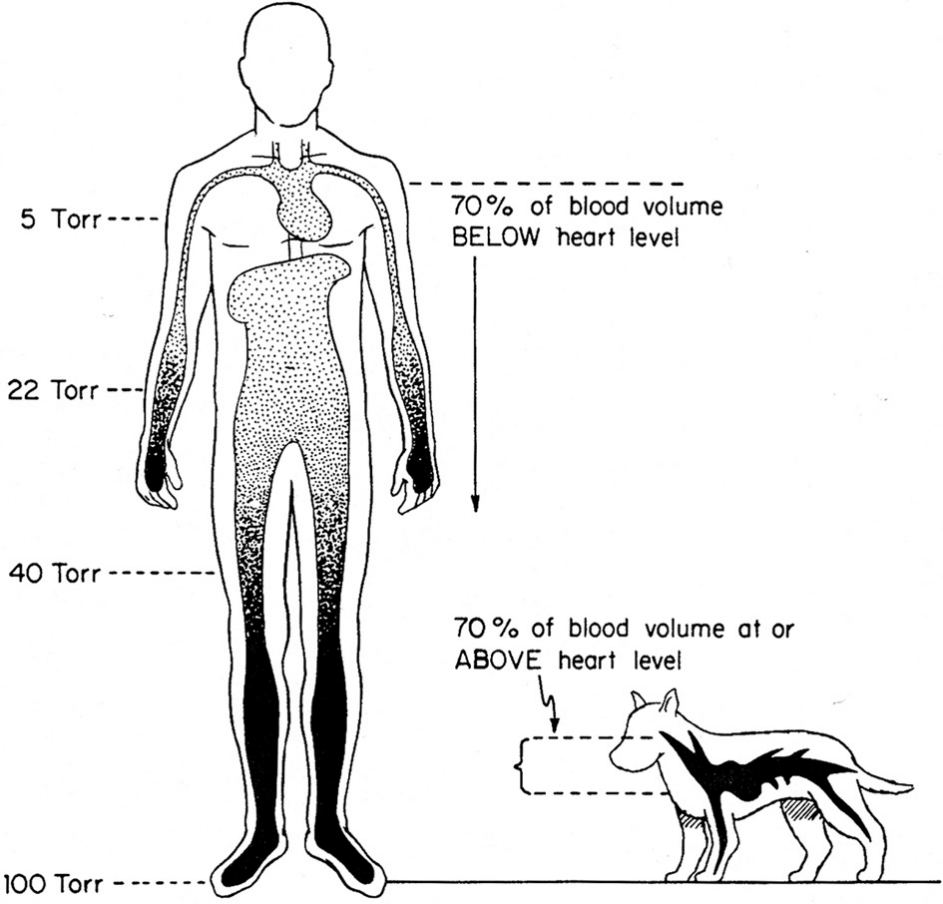
**Effects of terrestrial gravity on the cardiovascular system: the upright position**. The impact of microgravity affects 70 and 30% of blood volume in humans and dogs, respectively. From Rowell (1983).

Before the advent of the neocytolysis hypothesis, interesting data on RBC survival were obtained from studies in rats aboard the Soviet Biosatellite Kosmos 782 and 936 (Leon et al., 1978, 1980). Here, a cohort-labeling method was used by feeding the animals ^14^C-glycine 19 days before flight, and quantifying the respiratory ^14^CO, derived from heme catabolism, starting at day 2 from landing. Unlike the random labeling with ^51^Cr, this procedure is in principle immune from elution of the label, since only the ^14^C biosynthetically incorporated into heme is measured (Landaw and Winchell, 1970). A number of parameters can be calculated, including the rate of random cell death and the rate of cell death by senescence (Leon et al., 1980). It turned out that the rate of random cell death of RBCs was higher in rats during space flight with respect to both a control group on Earth and, most notably, a group of rats that were exposed to simulated 1 g gravity on an on-board centrifuge. This was the first piece of evidence pointing to weightlessness as the cause of RBCM reduction, and excluding a number of possible factors such as hyperoxia, radiation, forces associated with launch and re-entry, temperature and humidity, diet. An increased rate of random destruction, and the consequent decrease in RBC death by senescence, together with the fluid shifts and central plethora (that may occur also in rats, as hypothesized, but not verified, by the Authors of this work) suggested on the one hand that RBCM reduction resulted from accelerated haemolysis, not only from suppressed erythropoiesis and normal destruction rate, and on the other, that the fluid shifts could change splenic function leading to increased sequestration and haemolysis in this organ (Leon et al., 1980). Another hypothesis, brought on by Tavassoli (1982) was that intramedullary haemolysis or selective lysis of reticulocytes (neocytes?) could explain the data of Leon et al. on rats. On the other hand, random destruction, by definition, affects RBCs independently from cell age, and moves the focus on altered splenic function rather than specific cell properties. Because in these works on rats EPO concentrations were not monitored, an important parameter is missing, making it difficult to rationalize the data. Another important aspect is whether or not a plethora of the same magnitude as the one observed in humans also occurs in small animals exposed to microgravity (Figure 3).

A causal relationship was established (although only at a speculative level for the moment), between the disappearance of a young RBC subpopulation and the transient decrease in EPO observed during flight. It will be now examined on the basis of what experimental evidence this concept was extended to other models of physiological adaptive response and whether the causal relationship between neocytolysis and EPO decrease, which are so far only correlated events, was corroborated.

## Neocytolysis after descent from high altitude

Since, more than 60 years ago, Merino (1950), analysing erythropoiesis in people living at high altitude (4540 m a.s.l.), described a decreased production and an increased destruction of RBCs during the deacclimatization at sea level, these observations remained obscure until 2001, when results of a study were published supporting the notion that neocytolysis is triggered also on descent from high altitude.

In this study (Rice et al., 2001), nine men living at 4380 m a.s.l. and polycythaemic (as expected for people living there) were analyzed for a baseline period of 11 days at high altitude and for additional 9 days after descent. Five blood samples were taken at altitude and four samples at sea level for analysis of various haematological parameters: haematocrit, haemoglobin concentration, reticulocytes, serum EPO, serum ferritin, serum transferrin receptor and heme isolation. In this study, to differentiate between young and old cells a cohort-labeling method, different from the ^51^Cr random-labeling, was adopted. Participants ingested 1 g of ^13^C-glycine on day 1 of the study and 1 g of ^15^N-glycine on day 9. Blood and stool specimens were then analyzed for ^13^C and ^15^N enrichment in heme and stercobilin. Three participants were injected subcutaneously with 1200 U EPO daily beginning on the day of descent. Upon descent to sea level, serum EPO levels were decreased by approximately 80% in the six participants who did not receive exogenous EPO.

The average decrease in RBC mass was 7.0–9.6% within 3–7 days of descent. In the legend to the figure reporting those data, however, it is said that the measurements were performed “after 10 days at sea level.” (Rice et al., 2001). It must be considered here that a decrease of RBCM of 7–9.6% in 7 days is close to the physiological value of RBC destruction under conditions where RBCs are not replaced by new ones (EPO levels decreased in these subjects by an average 80%, much more than in space missions). Concerning the isotopic enrichment in heme and stercobilin, the latter could not be determined because of low isotope levels, whereas the former was reported as being compatible with a decrease in young red cells at descent, although the differences were not statistically significant. Therefore, the main argument of the paper in favor of young RBC destruction was based on the observed elevation of ferritin levels, which was interpreted as the transfer of iron to stores. However, ferritin saturation was not measured, and this, combined with the observation that the levels of circulating transferrin receptors did not change, raises the suspicion that ferritin might have been elevated for other reasons (see above the discussion on these two parameters in space missions).

Another piece of evidence presented as a strong argument in favor of a haemolytic process triggered by descent to sea level was the observation that reticulocyte counts did not change with respect to baseline values (120 ± 52 × 10^9^ cells/L at baseline vs. 141 ± 52 × 10^9^ cells/L during the first 6 days at sea level). Only on day 8 at sea level did the reticulocyte counts decrease to 90 ± 54 × 10^9^ cells/L, a delayed decrease that was correctly interpreted as the response to continued EPO suppression (remembering that reticulocytes released in the circulation at day 6 after descent were conceived in the bone marrow 6 days earlier in the presence of normal EPO levels). However, what is hard to reconcile with the neocytolysis hypothesis is that during the days when neocytolysis should be at its zenith, because plethora is at a maximum and EPO levels at the nadir, i.e., during the first week at sea level, the reticulocytes are spared by the process, as if they were something different from the neocytes. Yet, the Authors concluded: “that the decrease in red cell mass while reticulocyte production remained normal provides conclusive evidence of a haemolytic process” (Rice et al., 2001). Evidently, different features in the reticulocyte and neocyte must be invoked to accept this explanation, but a discussion about this aspect has never been conducted. Results of reticulocyte measurements in space missions (see above) and in descent from altitude are inconsistent and probably not informative of underlying changes in erythrokinetics. Mechanisms for the selective removal of RBCs produced under conditions of acutely enhanced erythropoiesis (stress reticulocytes) may also be invoked (Landaw, 1988) but this explanation, while possibly applicable to RBCs produced at high altitude under hypoxic conditions, does not obviously work for space anaemia, where neocytes that were produced before flight (i.e., under normal, physiological conditions) are supposed to be destroyed.

It should be also mentioned results of a research where, after a 9 day training at 1900 m a.s.l. and return to sea level of eight elite endurance cyclists, total reticulocyte counts were slightly decreased, whereas the population of immature reticulocytes (detected as high- and medium- fluorescent cells in the analytical procedure) appeared to better reflect the changes in RBC production and destruction before and after descent, with a pattern compatible with an undergoing neocytolytic process (with all the limitations of the study, which include short acclimatization period, relatively modest altitude and high variability of the data) (Nadarajan et al., 2010).

## Experiments on RBCs subsets

The experiments conducted *in vivo*, through determination of haematological parameters on high altitude dwellers or astronauts gave conflicting and incomplete results, partially due to reasons inherent to this type of experiments, including the small number of subjects examined, which prevent any robust statistical analysis, and the environmental constraints the approach imposes. Blood withdrawal in space or at high altitude can be uneasy; furthermore, the storage of samples in space or at high altitude, with limited laboratory facilities, could alter the properties of the blood components. Finally, a further bias of the systemic approach is the non-univocal interpretation of some haematological parameters whose alteration can be due to reasons different from altered RBC mass and erythrolysis, as mentioned above.

In an attempt to somehow circumvent this problem, we tried to shift from a physiological, systemic approach to one at the cellular and molecular level, by analysing blood samples drawn before and after the exposure to microgravity or hypoxia, and by separating RBCs by density into age-related fractions (Risso et al., 2007, 2008).

A previous validation of the density separation procedure (Risso et al., 2007), and a characterization of some membrane components differentially expressed by old red cells, suggested that the neocytolysis hypothesis could be challenged through the investigation of the cell numbers and properties of the density separated subsets. Due to the technical constraints described above, it was not possible to conduct parallel measurements of 4.1a/4.1b ratio (see below), to verify whether changes in cell density that could occur on exposure to altered physiological conditions affected a selected subpopulation of RBCs.

In both the astronauts and the mountain climbers groups, after the exposure either to microgravity or high altitude, some of the standard haematological and cellular parameters (decrease of EPO plasma concentrations, increase in ferritin, decrease in reticulocytes) related to erythrolysis, indicated that this process was under way. In all subjects of both groups the percentage of low density (neocytes) after *vs*. before (control blood samples) the exposure to hypoxia or microgravity was reduced. The expression of membrane components which decline (CD55, CD47) or are translocated to the outer leaflet of the membrane bilayer (phosphatidylserine) in aged RBCs, and seem to be involved in the positive or negative regulation of phagocytosis, indicated that the less dense red cells (the few surviving neocytes) had a senescent phenotype, making them more prone to ingestion by macrophages (Risso et al., 2007, 2008).

In mountain climbers indeed a dramatic shift of the whole RBC population to high density regions of Percoll gradients seemed to indicate lysis also of many RBCs of the middle density (middle aged) subset and suggested a generalized increase in density of the whole RBC population, raising some doubts on the age-density relationship, at least under certain circumstances.

The observations on RBCs drawn from people adapted to high altitude were extended to other biochemical features. The ATP content of RBCs isolated during the deacclimatization from hypoxia was significantly higher than that of the control populations. Furthermore, alteration in the membrane-skeleton was found by a proteomic analysis, i.e., fragmentation of spectrin and actin (Risso et al., 2010). Finally in an investigation on blood samples drawn from mountain climbers, over and immediately after a 17 days' time spent at 3100–5600 m a.s.l., we observed that high altitude induced the expression of foetal haemoglobin. Gamma globin expression was detected in low but not in middle or high density RBCs, when separated from samples drawn during and after exposure to hypoxia (Risso et al., 2012). These observations, if on the one hand seem to suggest that also low density RBCs separated from particular blood samples can be reasonably regarded as a “neocyte-enriched” subset, on the other hand indicate that the “neocytolytic process” over high altitude could be related not only to a reduced EPO synthesis and to the need to decrease RBCs mass, but also to their particular features. Under the hypoxic stimulus, erythroid precursors likely generate RBCs which are endowed with biochemical features more fit to hypoxia and which, upon return to normoxia, should be cleared.

## The role of EPO

The role of EPO is controversial, in part for the reasons exposed above, because if one accepts the idea that the decrease in RBCM observed in microgravity and on descent from altitude can be accounted for only by the physiological destruction of 1% RBCs/day under conditions of decreased EPO, then there would not be an active, cytoprotective effect of EPO on circulating erythrocytes. The magnitude of RBCM deficit does however appear to be larger than expected at least for space missions of short duration (7–15 days), especially because EPO secretion is not suppressed, but only decreased, and only temporarily. The “space anaemia” condition is in fact described in haematology textbooks and treatises but it seems that neocytolysis is not yet received unanimously as the explanation for this phenomenon, because the fluctuations in EPO levels occurring during space flights are not considered sufficiently large to affect the RBCM (Erslev, 2001).

Conversely, RBCM decline after descent from altitude appears to be less dramatic, and compatible with a physiological rate of RBC destruction under conditions of strongly decreased EPO levels (see discussion above). It is tempting to speculate that the decrease in RBCM occurring in space and on descent from altitude may only be apparently related to a common mechanism. At any rate, to account for RBCM decrease under both circumstances, a cytoprotective effect of EPO on circulating RBCs or RBC subpopulations has been invoked. The abrogation of any eryhrolytic process by EPO infusion in three mountain dwellers (the subjects remained polycythemic and no haematological parameter changes related to erythrolysis were observed), favors the hypothesis of an ongoing erythrolysis in the subjects that were not treated with EPO, and a possible causal correlation between EPO and lysis of (young) red cells (Rice et al., 2001), suggesting in that case a protective role played by EPO.

The existence of cytoprotective effects of this haematopoietic hormone on a variety of different cell types has been described (Kowalczyk et al., 2011; Shin et al., 2012). A study of the complex expression pattern of EPO receptors in primary human erythroid precursors induced to differentiate to reticulocytes, revealed the lack of EPO receptors, at least in these *in vitro*-differentiated reticulocytes (Sawada et al., 1990; Broudy et al., 1991; Wickrema et al., 1992). Mature erythrocytes were found to virtually lack EPO receptors, although murine RBCs, specifically the 2% youngest (less dense) RBCs, were found to express them (Mihov et al., 2009). In human erythrocytes, EPO has been described to inhibit a Ca^2+^-permeable cation channel, whose opening can be evoked *in vitro* by hyperosmotic shock, via a mechanism implying the binding of EPO to EPO receptors that were found to be present, as ^125^I-EPO-binding sites, in an average number of six per cell (Myssina et al., 2003). It must be observed, in the light of recent evidence (Minetti et al., 2013), that care must be taken in avoiding artefacts originated by contaminating granulocytes in RBC preparations, especially when a possible enrichment in contaminating cells is produced when RBCs are sub-fractionated according to density.

At systemic level, pleiotropic effects of EPO acting not only as a haemopoietic hormone but also in the regulation of PV, in interplay with the renin–angiotensin–aldosterone axis, have been reported (Lundby et al., 2007). On the other hand, EPO effects seem to be context-dependent, as EPO produced in brain, liver, spleen, lung and testis (where EPO mRNA was detected) is unable to substitute for renal EPO in chronic kidney disease, and brain EPO seems to act locally as neuroprotector (Jelkmann, 2011). Other difficulties in accepting direct effects of EPO, mediated by its receptor, in non-erythroid tissues include evidence that after suppressing EPO receptor expression in all organs except bone marrow in mice, normal and fertile animals develop (Suzuki et al., 2002), and that EPO receptors are undetectable in non-haematopoietic cells (Sinclair et al., 2010; Jelkmann, 2011). On the other hand, it was shown that erythropoietically inactive EPO derivatives act as cytoprotective factors in animal models (Leist et al., 2004). Would it be possible that EPO act, by molecular mimicry, on a different receptor system in other cell types, including the RBC? Against this possibility is evidence that the increased viability of peripheral RBCs measured with the ^51^Cr random labeling technique in uraemic patients on recombinant human EPO treatment was ascribed to molecular effects of EPO on the erythroid precursor cells in the bone marrow, and not on peripheral circulating erythrocytes (Polenakovic and Sikole, 1996).

Because of the inevitable scepticism with which claims of the expression of EPO receptors in erythrocytes are met, it could be hypothesized that the observed effect of EPO may be due to the interference of EPO with other molecular targets in RBCs.

## Conclusions

Neocytolysis is a physiological process which could shorten RBCs lifespan in response to a changed external environment and lead to a reduction in RBC mass. Despite many studies, the factors determining the lifespan of cells (including RBCs) that circulate in blood are not fully understood. In the past years, in red cells treated *in vitro* with some pro-apoptotic agents, a programmed cell death-like process has been described, which has been dubbed eryptosis (Lang et al., 2008). Investigations on the features and events associated with eryptosis have shown that they are reminiscent of those already described in old senescent red cells (dehydration, cell shrinkage, increase in cell density) and of those observed in nucleated apoptotic cells [PS exposure, increased intracellular Ca^2+^ concentration, altered functionality of ion channels, see Lang et al. (2012)]. Then it appears that, under some circumstances, RBCs death could be due to eryptosis.

Since RBCs lack the organelles and the multienzymatic, biogenic machineries able to protect the cells from external injuries, they are particularly sensitive to any changes either of inner proteins (altered haemoglobin, membrane lipid peroxidation, alteration of membrane-skeleton) or of external signals perturbing their homeostasis.

Within this framework, a shortening of lifespan is conceivable whenever changes in haemoglobin (for instance, in thalassemia or sickle cell disease) lead to alterations in membrane-skeleton, cell shape, redox conditions, or changes in the external environment requiring a fast reduction of RBCM, speed up the programmed death (or senescence process), followed by macrophage phagocytosis.

In this latter case, while reduction of RBCM has been documented in both exposure to microgravity and hypoxia, and some data seem to indicate that erythrolysis is not at random, two main issues need more detailed investigation: 1, the concomitant decrease in EPO levels and RBCM, since, although a causal link could be (and it has been) hypothesized, a formal evidence of this relation is still lacking; and 2, the identification of the targeted red cells. In relation to the latter argument, although the studies on age-related subsets seem to indicate that the low- / middle-density RBCs could be prone to phagocytosis, in view of their “eryptotic” (or “senescent”) phenotype, the relation between low density and age on the RBCs population after exposure to hypoxia or microgravity is questionable.

Neocytolysis is a fascinating hypothesis that, for the reasons exposed here, should be subjected to further scrutiny. It would benefit from being tested with additional methods that are immune from artefacts possibly arising from the different conditions existing on Earth and under microgravity. One such approach would be to conduct systematic and accurate measurements of the RBC age parameter 4.1a/4.1b, which is an absolute marker of RBC age independent on cell density, metabolic activity or imponderable side effects of radiolabeling. It is a molecular clock (Robinson and Robinson, 2004) whose ticking is only dependent on temperature, and therefore is relatively stable in a homeothermic animal. Measurements of 4.1a/4.1b could be performed on the total population of circulating RBCs or on cells separated by density. This approach yielded interesting results when applied to the study of how cell age impacts on RBC stored under blood bank conditions (Minetti et al., 2001). Moreover, a more refined and rigorous definition of the neocyte is required, because the reticulocytes are sometimes comprised in this definition (space missions in general) and sometimes not (descent form altitude). The difficulty in discovering (Chang et al., 2009) molecular species in neocytes which could mediate the response to decreased EPO levels, leading to recognition and destruction of young RBCs, may be a symptom of the extremely elusive nature of these species, depend on the necessity of implementing a multicellular *in vitro* model (Trial et al., 2001; Trial and Rice, 2004), suggest to look for off-target effects of EPO (Jelkmann, 2011), or indicate the very absence of such molecular machinery.

It would be worth restarting from what we know about RBCs of different age. For instance, how the intracellular ion homeostasis and membrane permeability are regulated in response to microgravity. Nothing is known of the calcium content or permeability of RBCs (Bogdanova et al., 2013) in space. Young RBCs have lower sodium and higher potassium and water content per-cell than older RBCs. There are several pathways that can be activated and determine potassium efflux (Bernhardt and Weiss, 2003). This could result in cell shrinkage, associated or not with membrane vesiculation (Ciana et al., 2004; Willekens et al., 2008), that could lead to reduction in deformability and the appearance of a senescent phenotype in otherwise young RBCs. A poorly deformable, dehydrated young erythrocyte would spend more time in the spleen sinusoids, where it could be recognized as abnormal and sequestered by the splenic macrophages. This is the rationale for the indication of splenectomy to increase RBC survival in some pathological conditions. “Nothing is known about splenic function in space” (Tavassoli, 1982), and it would be interesting to verify whether neocytolysis could take place also in splenectomized subjects. The possible occurrence of young RBC shrinkage under microgravity is compatible with the observation of the rapid decrease in MCV occurring in the 1st h of flight (see above). On the other hand, no study has ever been conducted on the impact of microgravity (in space or simulated) on RBC membrane permeability (Prof. Dr. Ingolf Bernhardt, personal communication).

Density separation of RBCs must be accompanied by determination of an absolute marker of cell age such as 4.1a/4.1b ratio. This will help clarify our own results obtained in mountain climbers after descent to sea level, where a massive shift in density profiles of RBCs from low to high density regions of Percoll gradients were observed (Risso et al., 2007).

Combined measurements of 4.1a/4.1b ratio and density separation of RBCs could also shed light on such discrepancies as the unclear behavior of reticulocyte counts, which were described to decrease during flight, but were also found 50% lower the day before flight (Leach and Johnson, 1984). Reticulocytes are probably not reliable indicators of the real size of the population of young circulating RBCs.

Further studies are needed to establish whether the reduction in RBCM, which is an established fact in a variety of blood disorders or physiological adaptive responses, is due, in each condition, to the removal of a selected population of RBCs, whether this is a population of “neocytes,” and what are the features of the targeted cells.

Given the complexity of the systemic responses, space anemia, uraemic anemia and deacclimatization anemia could be unrelated processes altogether. Each should be re-examined in its own context before generalizations could be made.

## Author contributions

Angela Risso conceived the review, conducted literature survey and wrote the first draft; Annarita Ciana and Cesare Achilli conducted literature survey, contributed to the introduction section and reviewed all other parts, Guglielmo Antonutto contributed to the literature survey and to discussions on the physiological issues of human haemodynamic and of red cells in space, Giampaolo Minetti conducted literature survey, wrote subsequent versions and overviewed the writing process.

### Conflict of interest statement

The authors declare that the research was conducted in the absence of any commercial or financial relationships that could be construed as a potential conflict of interest.
